# Exploring the Opportunities and Challenges of the Digital World for Early Childhood Services with Vulnerable Children

**DOI:** 10.3390/ijerph15112407

**Published:** 2018-10-30

**Authors:** Leona Harris, Niki Davis, Una Cunningham, Lia de Vocht, Sonja Macfarlane, Nikita Gregory, Saili Aukuso, Tufulasifa’atafatafa Ova Taleni, Jan Dobson

**Affiliations:** 1 E-Learning Research Lab, University of Canterbury, Christchurch 8041, New Zealand; leona.harris@canterbury.ac.nz (L.H.); lia.devocht@canterbury.ac.nz (L.d.V.); saili.aukuso@pg.canterbury.ac.nz (S.A.); 2 Child Well-being Research Institute, University of Canterbury, Christchurch 8041, New Zealand; nikita.gregory@pg.canterbury.ac.nz (N.G.); tufulasi.taleni@canterbury.ac.nz (T.O.T.); 3 Department of Education, Uppsala University, 75236 Uppsala, Sweden; una.cunningham@edu.uu.se; 4 Te Rū Rangahau: The Māori Research Laboratory, University of Canterbury, Christchurch 8041, New Zealand; sonja.macfarlane@canterbury.ac.nz; 5 Sector Enablement and Support, Ministry of Education, Christchurch 8013, New Zealand; jan.dobson@education.govt.nz

**Keywords:** internet addiction, mobile phone (or smartphone) use, young children, early childhood education, parenting, emergent bilinguals, intergenerational language transmission

## Abstract

Potentially addictive behaviours supported by the internet and mobile phones raise concerns in education services for early childhood. Although there is evidence that screen media can distract the attention of young children, there was a massive uptake of digital devices by early childhood centres (ECCs). We investigated practices of families (*n* = 85) and of six ECCs serving vulnerable children in New Zealand, many of whom are emergent bilinguals. Descriptions of the limited and exemplary choice of screen media of the ECCs include digital portfolios containing children’s learning stories in multiple languages illustrated with digital photos. This was facilitated by increasing partnership with the families and the inclusion of their languages in the physical and digital landscapes of the ECCs. However, these families and the ECCs are seeking additional guidance to face the complex challenges of the digital world. These early findings from our national research programme, A Better Start, E Tipu E Rea, already informed significant changes in the ECCs; we also identified the potential for young children to act as agents of change.

## 1. Introduction

Childhood today is increasingly technologically constructed [1] with digital technologies such as computers, tablets, and smartphones now commonplace in many education systems and homes worldwide [2]. While the ubiquity of these technologies has beneficial outcomes for youngsters’ learning and socialisation, it contributes also, among other detrimental outcomes, to the loss of minority languages and cultures [3]. This loss is of concern given the increasing body of evidence that bilingualism and multilingualism can have lifelong cognitive, health, and economic benefits (see, for example, Reference [4]).

Addictive behaviours supported by technologies, such as the internet and mobile phones, raise concerns, particularly in health services for early childhood. The bold claims of the American Academy of Paediatrics Committee on Education in 2001 included recommendations reducing the exposure of children under six years of age to screen media. These arose from concerns about overuse of digital devices, resulting in young children missing developmentally appropriate activities and/or not getting enough exercise; in other words, typical activities in childhood are displaced by the adoption of digital media. Evidence that media can distract the attention of young children increased since those recommendations were published [5]). However, directives to simply reduce time spent with digital devices are unhelpful and impractical in a world in which digital devices have become ubiquitous, and when a range of devices with apps and digital environments now marketed include some supporting developmentally appropriate activities that were adopted by early childhood educators and other experts in the field [2].

In addition to the concerns about the exposure of young children to digital media, concerns about the influence of digital media on the behaviour of family members emerged over the last decade. Given the complexity of effective parent and sibling interaction with young children [6], it is possible that increasing the use of mobile phones and other devices can adversely impact child development, particularly in families suffering from some form of media addiction.

The study documented in this paper was partly prompted by these concerns. It describes the linguistic landscapes, as well as the deployment of digital technologies within them, of six early childhood education (ECE) centres (ECCs) in New Zealand to explore how such centres contribute to young children’s language development. The provision of high-quality early childhood education was shown to have a beneficial impact on the development of young children, particularly those from low-income families [7].

We set our study within these centres because the children attending them are of an age (four to six years) when they are rapidly developing their language and social skills. We also wanted to gain a better understanding of how digital technologies might further support the evolution of linguistic landscapes in ways that provide a better start within education systems for young children, particularly those who acquire more than one language (emergent bilinguals; cf. [8]), and for whom an enriched linguistic landscape, visibly valuing their home language, has the potential to make the most impact [9].

New Zealand is, for several reasons, a particularly interesting location in which to explore the interface between digital technology use in ECE and the effort to support emergent bilinguals attending ECCs.

1. Opportunities and challenges linked with the digital world recently grew in New Zealand with the rollout of ultrafast broadband nationwide and the consequent expanded internet access in schools, a great many homes, community facilities, and workplaces [10]; from 2015 to 2017, the “frequency, range of activities, and the number of devices people use to access the internet all increased” [11].
2. New Zealand’s ECE curriculum, Te Whāriki [12], is grounded in the country’s founding document from 1840, te Tiriti o Waitangi (Treaty of Waitangi), which is highly respectful of cultural and linguistic diversity. Written in both Māori and English, Te Whāriki is based on four principles (empowerment, holistic development, family and community, and relationships) woven together with five strands (wellbeing, belonging, contribution, communication, and exploration). The term “oral language” used within Te Whāriki refers to any method of communication that “the child uses as a first language” (p. 42). The culturally safe development of children’s language skills is essential (e.g., Reference [13]).
3. New Zealand’s awareness of the lifelong benefits of bilingualism and multilingualism is relatively recent [14]. Māori did not become one of the country’s three official national languages until 1987 (the other two are English and New Zealand sign language), and despite New Zealand’s increasingly multilingual population, migrants are generally encouraged to adopt English at the expense of their heritage languages [15]. English remains the language most commonly spoken in New Zealand and is the de facto language of nearly all educational provision in the country.
4. New Zealand is recognised as a linguistically “superdiverse” country with over 160 languages, in addition to English and Māori, with Samoan, Hindi, and Northern Chinese more commonly spoken [16]. Te reo Māori words are included in this article in line with common practice.

We aimed to gather evidence in a way that spread good practice in regard to enhancing the linguistic landscapes in the ECCs and minimising the risks that come with the digital world.

## 2. Growing Up in a Digital World

Hsin, Li, and Tsai’s comprehensive review of studies on information and communications technology (ICT) use in ECE [17] found positive associations between children’s language and literacy development and their use of digital tools and toys, although the learning outcomes generally depended on how the children used the technology. Chaudron’s Europe-wide study investigating the digital technology behaviours of children (0–8 years) [18] showed children typically using digital devices on their own, with parental management restricted to limiting length and frequency of use. Parents had little knowledge of what their children were actually doing in the digital world, however. Aubrey and Dahl [19] found ECE educators likewise lacked detailed knowledge of children’s digital experiences, and that parents rarely shared information with educators about how their children were using digital technologies at home. Marklind and Dunkels examined the introduction of tablets by early childhood teachers in Sweden [20], sometimes in the face of opposition from colleagues or parents, to develop children’s literacy and school-oriented language development, which is especially relevant for multilingual children.

Adult and adolescent addictive behaviours relating to problematic mobile phone use (PMPU) [21], have been found to be “positively associated with stress, depression, sleep disturbances, extraversion, female gender, young age, and poor academic or professional competence or performance” (p. 1213) and to affect learning [22]. However, it remains important to be cautious when applying the label addiction using the connection with substance abuse as a way of categorising online behaviour as disordered [23]. In the context of early childhood, the poor competence or performance of particular concern is around parenting and the contribution of siblings to the healthy development of young children. Kildare and Middlemiss’s comprehensive literature review [24] identified that there was a range of both parenting benefits and complications with the integration of mobile devices in their day-to-day lives, suggesting that the impact on any particular child was wide-ranging and complex. The common themes that emerged included “parent’s level of absorption with their mobile devices, child safety in the presence of parents’ mobile distractions and parents conflicted attitudes regarding device use, and decreased parental responsiveness and sensitivity towards children while distracted.” (p. 590). Perhaps most interestingly, they note that children’s screen time increases with parents’ screen time [25].

The findings from these studies highlight the importance of adults (most notably parents and educators) as mediators of children’s learning via ICT, as well as role models. Research by McPake, Plowman, and Stephen [26] found that young children’s use of digital technology at home tended to expand children’s early communicative and creative experiences. They also observed that maximisation of this learning once the children were in more formal educational settings relied on educators’ knowledge of these home-based learning experiences (including digital), and on their ability to carefully and creatively help the children build on those experiences.

However, adults cannot be effective mediators if they do not know how children are using these technologies at home and in formal educational settings, and if they do not understand how that use might be influencing the children’s holistic (including linguistic) development.

## 3. Emergent Bilinguals

Recent findings in neuroscience and from longitudinal cohort studies indicate that young children who acquire more than one language and retain them into adulthood have significantly better lifetime outcomes [27,28]. Research also shows that children learn a new language more effectively when they continue to use and develop their heritage language [29], and that pre-schoolers develop more positive identities as learners and are more engaged in literacy activities when their ECE settings include their home cultures and experiences [30].

Increasing opportunities in ECE settings for young children to engage in their family’s languages and cultures is important not only for reasons such as these, but also because the children’s respective languages and cultures encapsulate their heritage and enhance their mana (a word used in Māori to denote power, prestige, acumen, and efficacy). However, while children’s ability to develop and retain more than one language is challenging in educational contexts that are largely monolingual [31], proficiency in their parents’ language is important for the children of migrants [32].

Dressler [33] found that, as children move around their educational settings, they draw conclusions about the relative importance of the languages evident in the signs and artefacts around them, because it is these that indicate what is socially supported within those places. Thus, the presence of signs in more than one language and of culturally based artefacts in ECCs prompts and supports the use of multiple languages, enabling children and adults to co-build multilingual language and social skills [9,34].

Early childhood educators can value and support children’s use of their heritage languages by working in partnership with the children’s whānau (families, including extended family members) to ensure the authenticity of these inclusions in the linguistic landscapes of the centres. Bridging language practices between home and educational settings through parental participation, such as being expert partners in bi-literacy development through the co-creation of dual-language texts, reinforces children’s bilingual language development, as well as building their self-confidence, and strengthening their bilingual identities and their English language competencies [35,36,37,38].

However, there is very little research into the duration of internet and/or mobile phone use in educational contexts that aim to provide holistic support for young children, including ECCs. The benefits for emergent bilinguals are particularly relevant for vulnerable children.

## 4. Materials and Methods

Our main research question was “How are ECCs contributing to the development of the languages of young bilingual children who are growing up in a digital world?” In addition, for whānau with children who have lower levels of oral language ability in English, (i) How long do children spend on digital media? (ii) What are whānau views on the importance of their child’s multilingualism?

The study presented in this article conformed to the provisions of the Declaration of Helsinki in 1995, revised in Edinburgh in 2000 [39]. Ethical approval was granted by the University’s committee for human ethics (number 2016/21/ERHEC). For this paper, we bring together two complementary datasets of vulnerable emergent bilinguals who grew up in one locality, at a time when the digital world blended into their physical world: (1) qualitative ethnographic case studies of the linguistic landscapes of six ECCs in 2016 and in 2017, and (2) a multidisciplinary survey of families that included some of the same children during their first year of primary school in the same locality (*n* = 85). It may also be important to note that this multidisciplinary mixed-methods study was conducted within our national research programme’s Vision Matauranga, which braids western scientific methods with indigenous kaupapa Māori principles [40]. The research was also associated with a multi-agency initiative called “You Matter to Us” that emerged to support young children in an area with some of the most complex challenges faced in this country [41].

These children were situated within three suburbs of a large city purposefully selected because of its cultural diversity and over-representation of low-income families. Around 22% of people in this area speak more than one language compared with 15.8% across the city [42]. The languages other than English most commonly spoken in this area are Māori and Samoan. The details of our approach and methods are provided in other publications [43,44]; thus, in this paper, we provide a brief summary along with details of the participants. We begin with the methodology used with the ECC before describing the survey methodology.

### 4.1. Case Studies of Six Early Childhood Centres

**Figure 1:**
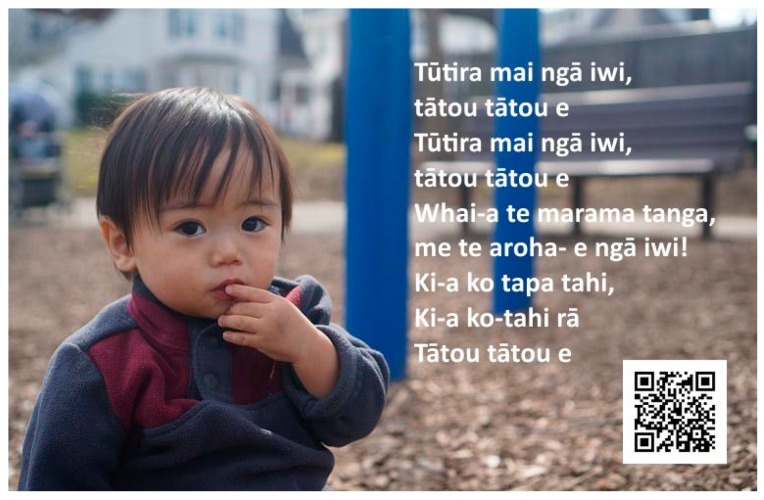
An example of innovative practice co-constructed with the pilot Māori early childhood centre (ECC) in response to parents’ need for support to sing along in the Māori language with their children at home [44]. The image of the child was retrieved from https://pixabay.com, which is released under the Creative Commons, as is this image CC BY SA3.

The first set of evidence was linguistic ethnographies [45] gathered in collaboration with six mainstream English medium ECCs. Reports from the national government department responsible for reviewing the quality of ECCs in New Zealand, called the Education Review Office, considered five of the ECCs “well placed’’ and the sixth ECC “very well placed” to promote positive learning outcomes for children (the highest two levels) [46]. To acknowledge the value of their participation, each ECC received a report of their linguistic landscape to use for their own purposes (plus advising edits). In addition, some development time was gifted on request. Figure 1 is derived from an image that was created as koha (a gift).

The ethnographic data for each linguistic landscape consisted of images in the form of still pictures, videos, and online screenshots. Still pictures and two videos (child height and adult height) captured the displays on the walls within each centre. Videos and screenshots (of publicly accessible pages online) were used to support interviews. Sixteen interviews were conducted with 18 staff (15 with individuals and one with a small group), as well as a few with whānau. In most ECCs, there was one interview with the head teacher and other teachers, and one interview with member(s) of a bilingual child’s whānau. In addition, there was one interview with the outreach librarian who provided library services for those with limited ability to visit community libraries. She visited many of these ECCs to promote the library services through storytelling, songs, and digital storytelling.

Linguistic content in the photos was classified by the language(s) visible. Selected artefacts (for example, the e-portfolio) were used to discuss each centre’s language and digital technology policies and practices, and to identify each centre’s engagement with whānau and the communities that supported the language development of its emergent bilingual children. The interviews were semi-structured and conversational, and questions about what they did to support the language development of their emergent bilinguals stemmed from the participants’ narratives and explanations of the physical and digital artefacts. The interviews elicited reflections on adults’ practices with emergent bilinguals and prompted ideas for future strategies. Interviews were transcribed and analyses were both inductive and deductive. Firstly, a short report was produced for the ECCs to verify and use for their own purposes, such as an annual review and future planning. Then themes around policies and practices relating to language and digital media were identified through coding references to the use of digital technologies and/or languages other than English. Other themes also emerged, such as teachers prioritizing social competencies, physical play, and the development of relationships over digital technology use, and the challenges faced engaging whānau when whānau were under increased pressures in everyday life. Expert researchers then validated the evidence for each theme, applying their expertise in ECE, linguistics, and/or the digital world.

### 4.2. The Survey of Families

Our overarching programme of research focuses on vulnerable children because our aim is to give them a better start [47]. As a proxy for vulnerability, we chose to focus on those with lower language levels. These children were in the first year of schooling in the same suburbs as the ECC and included children who previously attended the six participating ECCs. A questionnaire was developed to ask whānau (extended family) about the hauora (health and wellbeing), hononga (reading together at home), hinengaro (reading practices), and harikoa (positive identities) of their children, who were identified with lower levels of oral language ability.

**Figure 2:**
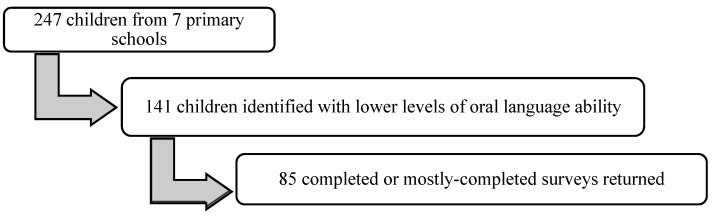
Visual representation of staged recruitment process of the whānau survey.

These children were selected from all 247 children in their first year attending the seven participating primary schools (see Figure 2). The screening employed two sub-tests from the Clinical Evaluation of Language Fundamentals Preschool, Second Edition (CELF-P2) [48], and the initial phoneme identity task from the New Zealand Computer-Based Phonological Awareness Database (CBPA) [49]. The 152 children who scored less than eight on the CELF-P2 subtest and/or less than six on the CBPA assessment were identified with lower levels of oral language ability.

All 141 whānau of those children with lower levels of oral language ability were invited to complete the questionnaire. Subsequently, 85 (60%) of these whānau questionnaires were returned, and the demographics of participants are shown in Table 1. There was unlikely to be much difference with those who did not complete the survey, because they had a similar demographic makeup to the 141 children identified with lower levels of language ability (see Table 1). Whānau completing a survey or interview were given a $15 New Zealand dollar (NZD) voucher for groceries to acknowledge the gift of their time. Various follow-up methods were employed to reach this number, including community workshops, one-on-one meetings (occasionally with a researcher who spoke their home language), phone calls, and emails. Only items related to multilingualism and the digital world are considered in this paper; another article will report in full on this survey [43].

## 5. Findings

### 5.1. Linguistic Landscapes of Six ECC

**Figure 3:**
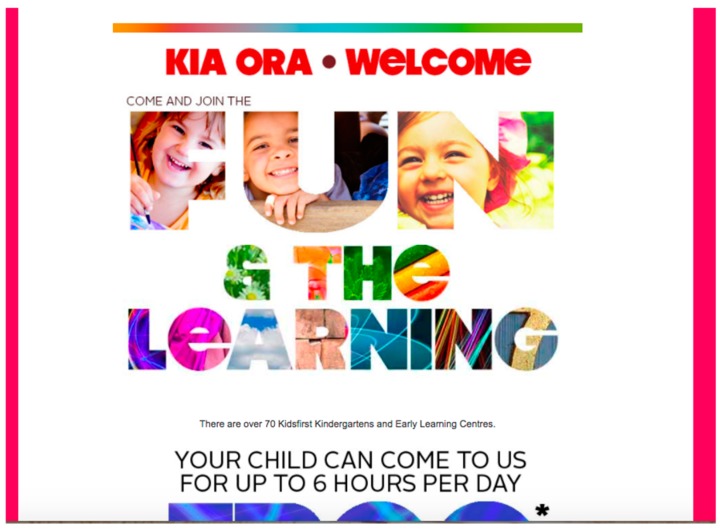
Screenshot of one early childhood education (ECE) centre website with bilingual welcome.

**Figure 4:**
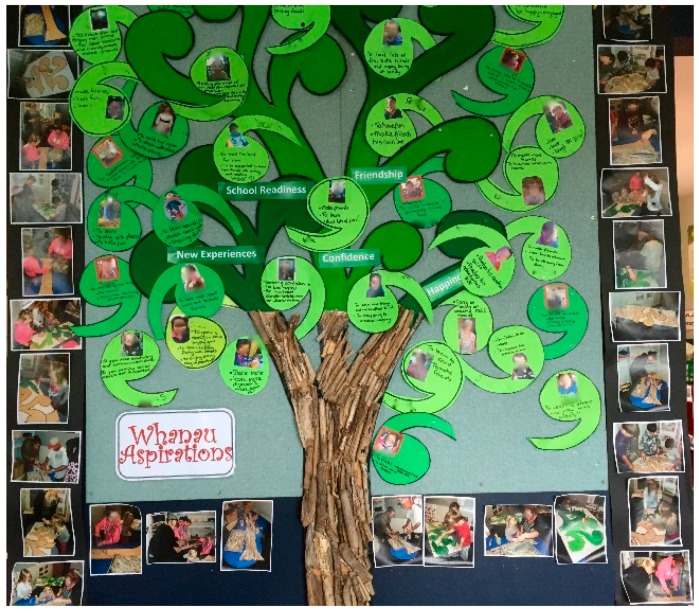
The Whānau Aspirations Tree echoes Māori culture. It was co-constructed by centre staff and whānau and set within the ECC’s main learning area. The photos were taken with a digital camera.

An overview of the languages that could be seen in the physical and digital landscapes of the six ECCs is followed by a discussion of themes relating to the perception and use of digital media, ending with a review of challenges. Table 2 provides a summary of the languages in the six ECE centres that was evident in their signs and artefacts. Of the 469 images taken of all wall displays across the six centres, 354 contained linguistic items. These signs and artefacts were categorised as containing only one language (e.g., English only), as having an equal presence of two languages (e.g., English and Māori), or as having the majority of one language and some presence another language (e.g., English with some Māori). This indicated that these centres were reflecting Te Whāriki curriculum guidelines relating to commitment and work within the bilingual (Māori and English) context of Aotearoa New Zealand. Other languages were also represented, with these typically responsive to the languages and cultures of the children attending the centres and purposively chosen to meet centre policies and practices, such as collaboration (see Table 2 and Figure 3 and Figure 4).

Most teachers interviewed said that the main purpose of the linguistic landscape was to engage whānau in their children’s learning and development. This engagement facilitated face-to-face conversations with whānau, making it easier to communicate centre aims and plans, discuss children’s activities, and promote conversations, collaboration, and language development. It was notable that all ECCs had a multilingual welcome near the entrance and online (see Figure 3).

Crucially, teachers emphasised that centres relied on whānau support and collaboration with respect to children’s bilingual language development. Multilingual languages were most commonly seen in basic greetings used in displays, e-portfolios, and written communication to welcome, respect, and celebrate culture, identity, and language. A Whānau Aspirations Tree was observed in most ECCs. The one illustrated in Figure 4 was particularly rich in languages and culture. All the children’s photos taken with a digital camera were place on the tree, an image that echoes Māori culture, and beside each photo was the family’s aspirations in their home languages that focused mainly on the social aspects. A copy was placed within each child’s e-portfolio. Pictures of the co-construction surrounded this large display.

After English, the Māori language, along with cultural symbols and references, was most prevalent with evidence in artefacts such as signs, labels, songs, commands, books, portfolios, and communications to the children’s whānau. The writing on the walls also served as reminders and prompts for the teachers to use languages with their emergent bilingual children. Pasifika languages were also used in songs, instructions, resources, and books that also contained cultural symbols and references; some were obtained through digital technologies such as iPads and computers.

The outreach librarian stressed the importance of supporting emergent bilinguals by creating an environment which welcomed the use of all children’s languages beyond the context of the child’s home by stretching across the contexts of the home and ECC through the child’s digital world. “It’s really interesting that [bilingual children] actually can’t translate for you and they usually won’t speak that language when they’re at preschool. It is out of context, this isn’t where that happens, [the bilingual children] are just not old enough to put that in place. [The digital world] is a huge part of who [the child] is and who their family is and it doesn’t just belong in that house, it can actually exist everywhere else.” (Outreach librarian interview, 2016).

#### 5.1.1. Blended and Digital Resources Were Used Purposefully

Table 3 provides an overview of the way in which physical and digital resources were commonly used in all ECCs. The key digital resource was the e-portfolio, often used alongside paper portfolios. The e-portfolio was used to collate children’s learning stories and compile observational records of children’s competencies and learning dispositions, as recommended in Te Whāriki. Portfolio content, which included digital photos, videos, and text in English, Māori, and the children’s home languages, was shared with centre colleagues, homes, and whānau residing elsewhere, and it provided an opportunities for whānau to contribute in their home languages. The portfolios prompted children to reflect on, share, and talk about their activities across contexts.

From one centre’s perspective, the e-portfolio had both benefits and disadvantages. One advantage to using the e-portfolio was the ability to connect, engage, and collaborate with family/whānau and a platform to use home languages. “In our learning stories and children’s [portfolio] books we’re really encouraging the partnership between whānau, the teaching team, and the kindergarten, so that we are encouraging them to contribute photos of things that are of cultural significance to them, things that are important to their family. So, photos are a really good way of doing that. We’re trying to use greetings as we are writing the story and maybe some images.” (Teacher interview, 2016). The disadvantage was that some parents chose not to access their computer until after their children went to bed; thus, the child missed the sharing experience. The teacher told us, “So, we’ve gone back to redoing them, we are still loading them electronically, but we are doing it in the book as well.” (Teacher interview, 2016).

All centres had a digital camera and used digital photos extensively, enabling teachers, children, and whānau to share experiences across environments and generate conversations regardless of language. Digital photos were displayed on walls, in portfolios (both types) on iPads, and on a large screen in the mat or seating areas. Each centre had one of these screens, and teachers played videos (recorded on the digital camera) of the children’s activities on it and occasionally multicultural stories and celebrations. Whānau could send digital photos via email to the centre or place them in the e-portfolios. Teachers often added text to the photos and asked whānau if they could translate the text into home languages. One teacher interviewed said, “I gave (the family) some photos of their two children, two boys, what they were doing, and then asked them to take them home and talk to the boys about those (photos). So, they’ve come back with some of their own dialect (written on them).”

Despite the ability to connect language and experiences across the children’s environments of home and the ECCs, there were concerns that digital media could be distracting. Teachers in all ECCs expressed concerns about the impact digital media may have that reduces the real-world experiences that children have, which generate the conversations of shared experiences that are necessary for language development. For example, “I don’t hear our children talk about those experiences…So if they are not talking about them to us, then I would guess that the parents aren’t talking to the children about doing them…So they might be doing them but there’s not a lot of language going on while they are doing them, (adults) are not mediating for (young children).” (Teacher interview 2016).

The outreach librarian also reflected that parents were not engaged with the literacy activities, as was hoped. However, she noted that many parents approached her asking about the apps she used during the story time session to download themselves. This response was different from that to physical books. She believed it was the instant accessibility and the desire for access to quality apps that was motivating (Researcher journal, 2016).

All the centres had iPads, used for games (in English), music, and accessing content stemming from the children’s interests. One ECC also had apps in Samoan and Māori. Centres carefully managed children’s access to iPads. For one centre, this entailed the use of a booking system that allowed the child 10 min of iPad use controlled with the input of a passcode. This system was self-managing, and the next child on the booking schedule would prompt the transfer of the iPad. At the time of the study, two of the centres were not actively using the iPads, due to technical issues. Neither prioritised funding for maintaining them, partly due to the difficulties in managing them, as well as the teachers’ stated belief that whānau preferred children to play outdoors and socialise with one another rather than spend time on screens.

Because all of the centres deemed (through policy and practice) the children’s emotional regulation and social competency a priority, their staff took care to ensure that digital technology use and associated language artefacts were meaningful to the children and likely to engage their whānau in supporting that child’s development. The majority of teachers thought that most of the children attending the centres had access to at least one digital device at home. If teachers knew that a whānau did not have home-based access, they typically encouraged them to use the free internet at a public library. However, and crucially, all teachers said they did not know how the children used devices at home and what content they could access on them, one said “I haven’t had any experiences [of children’s digital worlds]. I have to say it is not something that has been a passion for me or really interested me, so unless somebody is really struggling or there is a child that I haven’t spent time with and I think that the only time that I’ll be able to spend with them is on an iPad, then for me it’s not one of the top priorities.” 

#### 5.1.2. Issues and Access

There was concern about digital media distracting from young children’s social and physical development, particularly mobile devices, and a number of adults mentioned that controlling their use was problematic in all locations, including arguments at home among siblings. However, there was no mention of any “screen addiction”. However, fear of being judged, lack of shared information about how much is too much, and limited knowledge of such addictions may be why the term addiction was avoided. Alternatively, it may be less likely among families with low incomes. Indeed, in the previous year during the stakeholder meeting that informed the research design so that ECCs were included, one of the teachers passionately objected to the “Bring Your Own Device” policy that was being introduced in the neighbouring schools, because of the stress that it placed on families that already struggled to feed and clothe their children.

### 5.2. Survey Findings

As introduced earlier, the whānau survey enabled us to extend our findings relating to digital world and whānau perceptions of their children’s multilingualism. Despite the small sample size (*n* = 85) of the survey, there were some significant findings. The 85 questionnaires returned by whānau described the selected 50 boys and 35 girls with low language abilities attending their first year of schooling (age mean = 68.13 months, SD = 3.46 months) including many of the children that previously attended an ECC described earlier. Table 1 outlines the demographics of the children surveyed, as well as that for all the children with lower levels of oral language ability and the same nationwide demographics from the most recent census [42]. To aid in interpreting our results, we also compared our finds with the Growing Up in New Zealand (GUiNZ) cohort study of four year olds in 2013/2014 [50], while also noting that our findings are limited and not generalizable.

One of the questions in the survey asked, “During a typical week at home, approximately how many hours would your tamaiti/child/tama spend on digital media?”. In order to improve the cultural relevance of the survey, the questions inter-languaged English between Māori and Samoan words, e.g., “tamaiti/child/tama”. As shown in Table 4, we found that 6.8% of children did not engage with any digital media. When compared to the GUiNZ findings that 5% of four year olds did not engage with digital media, this suggests the proportion of cautious parents does not appear to be increasing, despite the increasing access to digital devices reported by the World Internet Project for New Zealand [11]. We also found that nearly one-third (32%) of children engaged with digital media for more than 5 h per week, equivalent to an average of more than 43 min per day. When comparing this result to GUiNZ data (41% of four year olds spending one or more hours a day on digital devices), our findings suggest that there is less use reported by whānau of these children than for the four year olds of the GUiNZ study. A limitation of our survey data was the way in which the question was asked and answered, and its variation with respect to the categories in GUiNZ survey, which also allowed open ended answers. These differences in survey design may be the cause for the differences in findings between our questionnaire and that in the GUiNZ study. As discussed later, any simple measure of time is problematic given the blending of physical and digital worlds.

Our statistical analysis to investigate potential relationships with this use of digital devices identified a two negative associations that were nearing significance: the number of hours on digital media and the whānau report of whether the child slept well through the night (*r* = −0.184, *p* = 0.122) and also with the whānau report of amount of sleep (*r* = −0.196, *p* = 0.09). Most of these children appear to be having the recommended 10–12 h of sleep (mean = 10 h 57 min, SD = 57 min), with only 4.9% having less than 10 h sleep per night. This result was similar to the hours of sleep per night (mean = 10 h 45 min) for four year olds in the GUiNZ study.

Whānau indicated that there were 54 multilingual children in this sample who spoke one or more languages in addition to English. In order to look into whānau perceptions, the importance of bi- or multilingualism was scaled from 0, not important at all, through to 3, very important. Counts are shown in Table 4; further analysis showed that whānau of multilingual children were the most affirmative. When looked at with reading practices, a positive correlation was found with children’s ability to read words that were pointed to (*r* = 0.315, *p* = 0.012). We also found that speaking more than one language was positively associated with children’s ability to read words that were pointed to (*r* = 0.308, *p* = 0.014), children’s ability to point and say words without being asked (*r* = 0.279, *p* = 0.027), and children’s ability to write their own surname (*r* = 0.471, *p* < 0.01). These findings may indicate the value for multilingualism in a child’s literacy development, such as richer language exchanges with whānau.

## 6. Discussion

Our descriptions of the linguistic landscapes of six mainstream ECE centres are reassuring in the way that they indicate purposeful integration of the digital world in the experiences of the children and the adults who support them, including the use of multilingual e-portfolios to share with whānau and to develop the children’s learning stories across time and space. Evidence from our survey also supported previous research indicating parental care of children. All the ECCs respected the children’s rights to access digital media and also controlled that access to ensure a healthy balance of activities in the knowledge that most children had a lot of exposure at home. This is exemplary, including the recognition of the need for further organisational and professional development.

Of particular value to multilingual children and their whānau, the teachers used languages other than English in the physical and digitally mediated linguistic landscape to welcome, respect, and celebrate the cultures and identities of all the children attending. The diverse languages presented in signs and artefacts also reminded and prompted teachers to use diverse languages with their emergent bilingual children. Teachers created most of the displays in the centres, especially the bilingual or multilingual ones, and this effort reflected their motivation for supporting emergent bilingual children.

Teachers and whānau prioritised social competencies, interaction, and emotional regulation as foundational for the development of their children’s languages. Centres carefully managed digital resources as an additional means of strengthening connections between the centre and home. Our later research showed that whānau engagement with the e-portfolio grew significantly from 2016 to 2017, along with the teachers’ skills and confidence in engaging with the software to collaborate on their children’s linguistic and other experiences. The linguistic landscapes nurtured reciprocal relationships with families and their communities to support children’s language development, including intergenerational transmission of language that is likely to result in a better start [36], particularly for the more vulnerable children who grow up with whānau speaking more than one language.

Incorporation of the digital world in the centres was not without concern, however, with teachers expressing interest in professional development and guidance in this area, particularly in terms of strategies that enable adults to share and shape children’s experiences in the digital world across both home and centre environments. Although early exemplars of learning stories to guide assessment practice in New Zealand ECE included resources such as digital cameras, email, and home languages [51], our study suggests that, as the complexity of the digital world increases, teachers and children’s families are likely to need more guidance to mediate that world for young children in ways that support both the children’s linguistic and holistic development.

While centres found it relatively easy to share children’s learning stories with the children’s whānau by encouraging them to add images and translations to the children’s e-portfolios, development of these portfolios created challenges for teachers, such as increased workload. The portfolios also highlighted issues relating to digital equity. For example, lack of technology or data to access e-portfolios at home reduced the opportunity to work with ECC staff to support their child. This lack of access could also potentially compromise the cultural safety interface between an ECC and homes [52].

Of most concern is the widespread knowledge that many young children are overexposed to digital media outside ECCs. Paudel et al.’s systematic literature review [53] suggests that children growing up in families with addictions such as those related to mobile screen media are at most risk and identified the following correlates: “older children, children better skilled in using mobile screen media devices, and those having greater access to such devices at home and whose parents had high mobile screen media use were more likely to have higher use of mobile screen media devices.” According to Lindenberg et al. [54], who identified prevalence rates of over 5% among adolescents in Germany, the World Health Organisation recently listed gaming disorder in addition to internet use disorder, indicating that the rising rates of various types of overuse of screen media are an increasing cause of health concerns. Our study updates our understanding of the protective factor of quality ECE [7]. Our study also raises the possibility that ECCs can guide and support families coping with these addictive behaviours to reduce that impact on these young children.

The writing of this article prompted us to consider ways in which to guide ECC. Concerns about the influence of digital media on the behaviour of family members emerged over the last decade. For example, Raman et al., whose detailed study [55] of screen exposure during young children’s daily routines explored the risk of having social-emotional delay, showed that children were exposed to screens throughout daily routines that would typically involve face-to-face interaction and conversational turn-taking, such as eating and sleeping routines. This persistent exposure to digital screens has the potential to distract attention away from the daily conversational interactions between adults and young children, which are necessary for healthy early development. Several teachers were concerned that parents were not mediating the use of digital media for their children, with one teacher saying that there was “not a lot of language going on”.

Digital distraction may cause these and other conversations to become shallow, thus reducing young children’s opportunities. Given the complexity of effective parent and sibling interaction with young children [6], it is possible that the increasing use of mobile phones and other devices can adversely impact child development, particularly in families suffering from some form of media addiction. For example, the Tizard and Hughes study [56] of the interactions of four-year-old girls with their mother at home in the 1980s contradicted the Piagetian thinking, which was prevalent at the time of their research, that the young child is illogical or whimsical (p. xiv). Instead, they concluded that children have an intense need to understand the world, which is reflected in the many “why” questions they asked at home (averaging 26 questions per hour). The analysis also showed that the turn-taking of the children and their mother supporting them to ask questions in a persistent and logical way in order to extend their understanding.

This unexplored relationship between digital distraction and young children’s need for interaction with parents indicates the need for additional research. It is also worth noting that Raman et al.’s methodology [55] clarified our understanding of how children experience the impact of the digital world for themselves and their whānau. This impact is spread across the entire day, from waking up until going to sleep, so that it becomes misleading to describe it in a unit of time, such as the 2.1 h from the GuiNZ study [50]. It will be important to apply this to the interpretation of the literature and in the design of future research, policy, and guidance that we share on our web sites (see Supplementary Materials).

## 7. Recommendations and Conclusions

The invitation to write this article in response to the IJERPH special issue on “Internet and Mobile Phone Addiction: Health and Educational Effects” led us to reflect deeply on that topic, and we now plan to apply this to our mission. The mission of our national programme of multidisciplinary research includes a strand on mental health within which Merry and her colleagues are developing e-health interventions, e.g., Reference [57]. “To predict, prevent, and treat vulnerability in obesity, poor literacy, and mental health through research excellence that will achieve healthy, well-adjusted, and well-educated tamariki and young people. We aim to achieve our mission by taking both a life-course and a “braided river” approach to integrate themes, research disciplines, and both western and indigenous models of knowledge and practice, as well as incorporating the use of digital technology into our proposed solutions” [47].

We identified the need for additional research into the effects of addictions related to the internet and mobile devices. In particular, there is a need to provide more nuanced guidance for parents and siblings so that these young children have more opportunities for deep learning of languages, concepts, and social behaviour. Such research could identify mitigating strategies to include policy guidance for health and educational services, an action that we previously recommended within our national “Better Start” programme of research.

Drawing on the findings presented, this policy guidance is recommended to include strategies that enable adults to share children’s experiences in the digital world. Vaughan and Beers’ account of iPad-related professional development for ECE teachers [58] could be adapted to integrate strategies that benefit emergent bilinguals.

Improvements in software and interface design are also needed. Miller and Kocurek’s five principles for the design and development of educational games for children under five years of age [59] could apply equally well to the development of digitally based multilingual activities and artefacts: (1) developmentally appropriate content; (2) reference to theoretical frameworks from the learning science field; (3) embedding learning in socially rich contexts; (4) ensuring diversity of content; and (5) creating a balance between play and real-world learning opportunities. To these five principles, we would add (6) opportunities for adults to engage alongside the children in their digital world(s) and to use all their languages.

We also have a recommendation with regards to multilingualism and the intergenerational transfer of languages. In Aotearoa New Zealand, Māori is recognised as a national language, and the ECE curriculum promotes its use as a living language. We found that it was often present in the six ECC landscapes as a language additional to English. Similar approaches could be extended to support the over 160 other languages in this linguistically “superdiverse” country [16], particularly for the more commonly spoken Samoan, Hindi, and Northern Chinese. Because the wider ecosystems influence the practices within ECE centres and homes [60], we recommend that policy documents, curricula, and related web resources be improved by weaving into them relevant languages and cultural images to support the enrichment of linguistic landscapes. Increased guidance and professional development is also recommended to strengthen language practices in ECE so that they are inclusive of linguistically diverse children. This approach was one that whānau and the community members who participated in our study and related workshops identified and endorsed.

Our future work includes the following commitments: (1) to expand our collection of evidence to describe changes in the landscapes in the first year of school in our target area; (2) to disseminate research-informed guidance for policy-makers locally nationally and globally that addresses the complexity of the digital world in which young children are growing up, including the support of intergenerational languages transmission. We also plan to further align our research with the “You Matter To Us” (YMTU) consortium which is exploring how inter-agency action combined with community development can influence the evolution of organisational policies and practices (system change) to enhance wellbeing of children aged 0–5 years.

### Conclusions

This research indicates that vulnerable children in quality ECC do have a better start in the digital world. ECE is improving multilingualism in Aotearoa New Zealand through its Te Whāriki curriculum, which ECCs are applying to evaluate and develop such practice. This practice limits “screen time” while also promoting strategies that deliberately deploy the digital world to serve the purposes of ECE in appropriate sociocultural ways. In addition to improving the quality of engagement, ECC staff deployed the digital world to increase the range and strength of their partnerships with whānau. As Taylor et al. [36] found, such purposeful strategies leverage the diverse languages of whānau with cultural support for their children, so it is also likely to have a positive impact on these families and their minority communities, which may result in a virtuous circle back to support all the children in the ECCs. In our research, the Whānau Aspirations Tree increased the sense of belonging, participation, and safety; mostly, it gave a strong presence in the ECC of the whānau voices. Given the fact that the area of the city in which the ECCs were located included one of the three most deprived suburbs in the country [61], this could have increased participants’ feelings of safety. Multiple sources of evidence evaluated for the related community project “You matter to Us” indicate that such improvements are possible [41].

Therefore, there is evidence that ECCs can contribute to the resilience of vulnerable children and given them a better start in the digital world. The increased thoughtfulness of the adults and siblings of young children is also likely to lead to increases in resilience to potential internet and mobile phone addictions. For example, United States guidance [62] (p. 77) recommends media mentors to guide children and states, “As the primary role models for technology and media use, adults should be aware of and set limits on their own technology and media use when children are present, and focus on children having well-rounded experiences, including moderate, healthy media use.”

The actions of these ECCs to deploy the digital world purposefully with developmentally appropriate sociocultural strategies included partnership with families. Projects by the World Organisation for Early Childhood Education (OMEP) [63] provide evidence that young children can act agents of change in their families to promote health and wellbeing. In the future, young children and their ECCs may be recognised as change agents redressing the challenges of addiction that are associated with growing up in a digital world.

## Figures and Tables

**Table 1:** Demographic information of the 85 children whose whānau participated in the survey compared with national demographics for children of the same age. NZ—New Zealand.

Characteristic	Surveys Returned (%) (*n* = 85)	Children Who Entered School with Lower Levels of Oral Language Ability (*n* = 141)	Percentage Nationwide of Children Aged 5–6 Years of Age in 2013 [42]
Gender			
Male	50 (58.8%)	49.60%	50.90%
Female	35 (41.2%)	50.40%	49.10%
Ethnicity			
NZ European and other ethnicity	48 (56.5%)	54.50%	62.80%
Pasifika	15 (17.64%)	21%	8.80%
Māori	22 (25.9%)	24.50%	20.10%

**Table 2:** Pictures containing linguistic items across the six early childhood education centres (ECCs) classified on the basis of the language(s) they contained.

Language(s) in the Items	Number of Items (*n* = 354)
English only	171
English with some Māori	43
English and Māori	50
Māori with some English	30
Māori only	22
Samoan only	10
Samoan and English	9
English/Samoan/Māori	8
Multilingual	11

**Table 3:** Representative sample of resources commonly observed in the six ECCs, classified by purpose(s) and an illustrative use. Figures refer to the examples of ECC artefacts.

Resource	Example of Use
Physical resource	
Whiteboards	Multilingual greetings and sentences in Māori and other languages (Figure 3)
Paper portfolio	Multilingual greetings and learning stories with Māori words and concepts
Labels	Prompt for teachers to use languages and welcomes
Cultural protocols	Normal practice in ECC, e.g., songs and prayers; mostly sung in Māori and Samoan (Figure 1)
Displays, national events	Resources supporting Samoan language week, Diwali Festival, etc.
Digital/blended resource	
E-portfolios	Children’s learning stories included multilingual greetings, Māori words and concepts, and family languages (e.g., Educa ePortfolio)
iPads	Cloud-based games supporting curriculum carefully managed; most apps in English
E-newsletters	Instead of or complementing paper version, these included multilingual greetings, and Māori words and concepts
Email	Used to send newsletters and photos to family (Figure 1)
Texting	Used occasionally for quick communication with families (e.g., to update a parent on their child)
Facebook	Produced by national organisations for their ECC; one had multilingual greetings
Websites	Produced by national organisations for their ECC; one had multilingual greetings (Figure 3)
Digital cameras	Extensive use by teachers for displays and artefacts to take home (Figure 1 and Figure 4)

**Table 4:** Frequency table of hours spent of digital media and of the importance of bilingualism.

		Frequency	Percent	Valid Percent
Hours on digital media per week	Never	5	5.9	6.8
	Less than an hour	11	12.9	14.6
	1–3 h	18	21.2	24.3
	3–5 h	16	18.8	21.6
	More than 5 h	24	28.2	32.4
	Sub total	74	87.1	100
Missing		11	12.9	
Total		85	100	
Importance of bi- and multilingualism	Not at all important	17	20	20.5
	Not that important	24	28.2	29.9
	Quite important	22	25.9	26.5
	Very important	20	23.5	24.1
	Sub total	83	97.6	100
Missing		2	2.4	
Total		85	100	

## Supplementary Materials

The Better Start research programme and EBinDW strand have web sites: http://www.abetterstart.nz/ and https://ebdwwebsite.wixsite.com/ebdw.
